# Functionally distinct alpha components are differentially modulated by attention and affect behavior

**DOI:** 10.1162/IMAG.a.1335

**Published:** 2026-08-17

**Authors:** Gamze Bilgen, Elie Rassi, Alma ElShafei, Julio Rodriguez-Larios, Saskia Haegens

**Affiliations:** Donders Institute for Brain, Cognition, and Behaviour, Radboud University, Nijmegen, The Netherlands; Center for Mind/Brain Sciences, University of Trento, Rovereto, Italy; Department of Psychology and Centre for Cognitive Neuroscience, University of Salzburg, Salzburg, Austria; Brunel University London, London, United Kingdom; Department of Psychiatry, Columbia University, New York, NY, United States; Division of Systems Neuroscience, New York State Psychiatric Institute, New York, NY, United States

**Keywords:** brain oscillations, alpha rhythm, ICA, working memory, attention, MEG

## Abstract

Alpha oscillations (~10 Hz) have been hypothesized to gate information flow in the brain by inhibiting task-irrelevant and disinhibiting task-relevant processing. Recent work using independent component analysis (ICA) showed that functionally distinct alpha components co-exist during a working-memory task. Here, we recorded magnetoencephalography (MEG) while participants performed a visual delayed match-to-sample task with pre- and retro-cueing to investigate the gating role of alpha and the impact of top-down attention on these dynamics. Consistent with prior findings, we observed that alpha power temporally tracked the presentation of the informative cue. Using ICA, we replicate previous results regarding the co-existence of two distinct alpha components (Alpha1 and Alpha2), and extend these findings by showing that these alpha components are differentially modulated during the decision delay with opposite relationship to behavioral outcomes. Furthermore, we report on preparatory mechanisms involving Alpha1 and Alpha2 and show that their subsequent impact on task performance varies with cue informativeness and the task relevance of upcoming input. When task relevance was unknown (i.e., in the retro-cue condition), increases in Alpha1 power before task-relevant input were associated with poorer performance. In contrast, when task relevance was known (i.e., in the pre-cue condition), pre-stimulus increases in Alpha2 power before task-irrelevant (i.e., to-be unattended) input was associated with better task performance. Combined, these findings are in line with our hypothesis that Alpha1 rhythms modulate visual processing while Alpha2 rhythms modulate the short-term storage of visual information. Crucially, these distinct (anticipatory) alpha dynamics only became evident using a decomposition approach, emphasizing the necessity of analysis methods that can disentangle overlapping rhythmic dynamics that stem from different sources.

## 1 Introduction

From the first description of the alpha rhythm (8–14 Hz) by Hans Berger (1929), its role in brain function has been extensively studied. Initially, the alpha rhythm was considered to reflect cortical idling or a state of rest (Adrian & Matthews, 1934; Pfurtscheller et al., 1996). However, the cortical idling argument was challenged by work suggesting that alpha regulates cortical excitability to gate information processing (Jensen & Mazaheri, 2010; Klimesch, 2012; Klimesch et al., 2007; Palva & Palva, 2007). In this view, decreased alpha activity facilitates information processing, while increased alpha suppresses interference, thereby controlling the flow of information. Studies adopting working-memory and attention paradigms have shown alpha power decreases in task-relevant regions and increases in task-irrelevant regions across visual (ElShafei et al., 2022; Sauseng et al., 2005; Thut et al., 2006), auditory (Weisz et al., 2014) and somatosensory (Haegens et al., 2010, 2012) systems. Critically, intracranial recordings in both human and non-human primates show a negative correlation between neuronal excitability and alpha power (Haegens, Nácher, et al., 2011; Haegens et al., 2022; Iemi et al., 2022). Furthermore, alpha activity was found to be positively correlated with working-memory performance in task-irrelevant regions (Haegens et al., 2010; Yu et al., 2017), whereas it is negatively correlated in task-relevant regions (Jiang et al., 2015; van Ede, Niklaus, et al., 2017).

Anticipatory alpha power is modulated by the locus of attention in visual (Kelly et al., 2006; Sauseng et al., 2005), auditory (Weisz et al., 2014), and somatosensory paradigms (Haegens, Händel, et al., 2011), and considered to play a key role in shaping the neuronal state of a region in preparation for upcoming input. Further supporting this view, previous studies revealed that alpha power increases before the anticipated onset of task-irrelevant information (Bonnefond & Jensen, 2012; Haegens et al., 2012; Payne et al., 2013; Snyder & Foxe, 2010; van Diepen & Mazaheri, 2017; Wang et al., 2019). However, some recent work does not report such alpha activity modulations related to task-irrelevant input (Jensen, 2024; Noonan et al., 2016; Van Moorselaar & Slagter, 2019).

One possible explanation for inconsistencies in the literature on anticipatory alpha activity is the existence of multiple alpha sources, which univariate sensor-level analysis cannot accurately capture. Previous studies that spatially separate different neural sources within the alpha band support the existence of multiple alpha components in attention (Sokoliuk et al., 2019) and visual perception paradigms (Benwell et al., 2019; Gulbinaite et al., 2017). Disentangling overlapping brain rhythms, possibly originating from different generators operating within the same band, is crucial to revealing the complete story of anticipatory oscillatory dynamics. Recent work suggests using decomposition methods, such as ICA, to differentiate posterior alpha contributions with distinct spatiospectral characteristics and potentially distinct functional implications. Applying this approach to a visual working-memory paradigm allowed Rodriguez-Larios et al. (2022) to separate one posterior alpha source that increased in power from another that decreased in power during memory retention.

Building on our recent work that further supported the gating role for alpha in information processing (ElShafei et al., 2022), the present MEG study aims to investigate how top-down attention impacts gating mechanisms. Participants completed a visual delayed match-to-sample task, which included cues indicating the to-be-matched stimulus either before (pre-cue) or after (retro-cue) the presentation of two sample stimuli, with probe timing either fixed (early/late) or jittered. This design allowed us to investigate the attentional modulation of anticipatory alpha activity based on the task relevance of the upcoming stimulus, as well as the temporal dynamics of top-down modulation in response to varying probe presentation times. By using decomposition methods to differentiate alpha rhythms from distinct sources, we aimed to disentangle the role of alpha in optimizing information processing through preparatory mechanisms.

## 2 Materials & Methods

### 2.1 Participants

Participants were 28 healthy right-handed adults (15 female, 13 male; mean age 26.8 years; range 21–37) without neurological or psychiatric disorders, who reported normal hearing and normal or corrected-to-normal vision. The study was approved by the local ethics committee (CMO 2014/288 “Imaging Human Cognition”) and in accordance with the Declaration of Helsinki. Participants gave written informed consent and were remunerated for their participation (10 euro/h).

### 2.2 Visual working-memory task

**Fig. 1.**
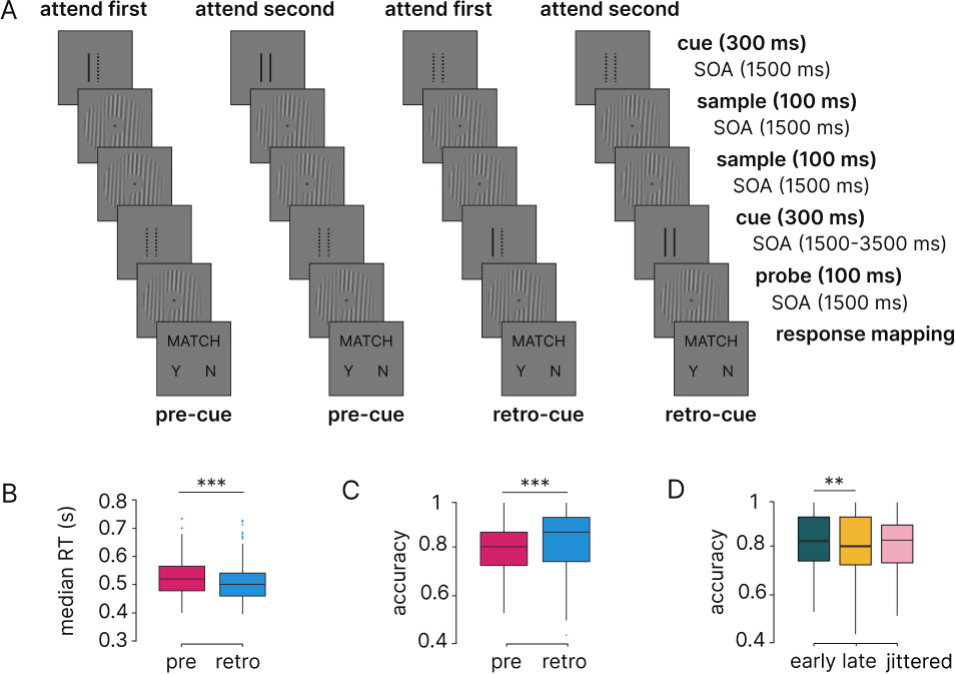
Paradigm and behavioral results. (A) Schematic of the four trial types: attend first sample (indicated by cue consisting of one solid line) or attend second sample (two solid lines) for pre- and retro-cue conditions (first vs. second cue is informative; uninformative cues consist of two dotted lines). (B) Participants were faster and (C) more accurate in the retro-cue condition than the pre-cue condition. (D) Participants were also more accurate when the probe occurred at a fixed early onset than at a fixed late onset. Within each boxplot, the horizontal line represents the median, the box delineates the area between the first and third quartiles (interquartile range). **p < 0.01, ***p< 0.001.

Our choice of task and stimuli was based on our previous study investigating the role of neural oscillations in working memory (ElShafei et al., 2022). Here, we used a delayed match-to-sample working-memory task where participants were cued to compare one of two presented samples with a probe stimulus and had to indicate whether the cued sample was of the same or different spatial frequency as the probe (Fig. 1A). The informative cue indicating which sample to attend was either the first cue (pre-cue condition) or the second cue (retro-cue condition). Each trial contained six main events: a visual cue followed by two visual samples, a second visual cue, a probe, and a response mapping diagram. All events except the probe were presented at a fixed stimulus onset asynchrony (SOA) of 1.5 s. Per block, the probe was either presented at a fixed early (1.5 s), fixed late (3.5 s), or jittered (1.5–3.5 s) SOA. The inter-trial interval (ITI) was jittered between 2 and 2.4 s.

We used a two (informative cue: pre- vs. retro-cue) by two (relevant sample: first vs. second) by three (probe onset: early vs. late vs. jittered) factorial design. Participants were cued to compare the probe’s spatial frequency with the first sample in half of the trials and with the second sample in the other half. They were informed about the relevant sample by a cue that occurred either before (pre; 50%) or after (retro; 50%) sample presentation. These four trial types were randomly interleaved such that within each block participants performed all four trial types. The different probe SOAs (early, late, and jittered) were presented in separate blocks (two blocks early, two blocks late, and four blocks jittered). Once presented with the response mapping diagram, participants gave their response (“match” or “no match”) by pressing a button with their right index or middle finger. The response-button mapping changed on a trial-by-trial basis in order to avoid motor preparation confounds in our decision-related analyses.

Participants first performed a few short practice sequences of the task (20 trials each) in which feedback was provided on a trial-by-trial basis, with green and red fixation dots for correct and incorrect responses, respectively. During the main experiment, we recorded MEG while participants performed 8 blocks of 32 trials each (for a total of 256 trials), in which feedback (percentage of correct responses) was provided on a block-by-block basis. Afterward, participants underwent a block where they passively viewed the gratings presented during the main block. During this (localizer) block, participants viewed 300 gratings with 1.5-s SOA. The full recording session lasted around 90 min.

### 2.3 Stimuli

A bull’s eye (outer black ring = 0.5° × 0.5° degree of visual angle (dva), innermost black dot = 0.25° × 0.25° dva) was presented at the center of the screen as the fixation point. Participants were instructed to always maintain fixation, and not to blink during the presentation of the stimuli. Each trial started with a cue presented for 300 ms. Next, the first sample consisting of an oriented grating (Michelson contrast: 40%, spatial frequency: 1 cycle per °, orientation: vertical, randomized spatial phase) was presented for 100 ms. The gratings were shown in an annulus (inner radius = 1.5°, outer radius = 7.5°, contrast of the stimuli decreased linearly to 0 over the outer and inner 0.5° radius of the annulus) around the central fixation (Fig. 1A). After an SOA of 1.5 s, a second grating was presented (100 ms), followed by a second cue (300 ms) and, after the variable SOA, the probe (100 ms), which, similar to the sample, consisted of an oriented grating. Finally, the response mapping was presented. The cued sample’s spatial frequency feature matched that of the probe grating in 50% of the trials. The experiment was programmed with PsychtoolBox (Brainard, 1997) in Matlab (Mathworks, Inc.).

### 2.4 Data acquisition

Stimuli were displayed on a semitranslucent screen (1920 × 1080-pixel resolution, 120-Hz refresh rate) back projected by a PROpixx projector (VPixx Technologies). Whole-head MEG data were acquired at a sampling frequency of 1200 Hz with a 275-channel MEG system with axial gradiometers (CTF MEG Systems, VSM MedTech Ltd.) in a magnetically shielded room. Six permanently faulty channels were disabled during the recordings, leaving 269 recorded MEG channels. Three fiducial coils were placed at the participant’s nasion and both ear canals, to provide online monitoring of participant’s head position (Stolk et al., 2013) and offline anatomical landmarks for co-registration. Eye position was recorded using an eye tracker (EyeLink, SR Research Ltd.). Upon completion of the MEG session, participant’s head shape and the location of the three fiducial coils were digitized using a Polhemus 3D tracking device (Polhemus, Colchester, Vermont, United States). Anatomical T1-weighted MRIs were obtained during a separate session. To improve co-registration of the MRIs and MEG data, earplugs with a drop of vitamin E were placed at participant’s ear canals during MRI acquisition. These anatomical scans were used for source reconstruction of the MEG signals.

### 2.5 Behavioral analysis

The influence of cue condition (two levels: pre- and retro-cue), relevant sample (two levels: first and second sample), and probe onset (three levels: early, late, and jittered probe) on median reaction time (RT; including correct responses only) and accuracy (percentage of correct responses) was tested using a linear mixed-effects model using the lme4 package (Bates et al., 2015) for R (R Core Team, 2014). For post-hoc analysis, we used the Lsmean package (Searle et al., 1980) where p-values were considered as significant at p < 0.05 and adjusted for the number of comparisons performed (Tukey method).

### 2.6 MEG preprocessing

MEG data were preprocessed offline and analyzed using the FieldTrip toolbox (Oostenveld et al., 2011) and custom-built MATLAB scripts. The MEG signal was epoched based on the onset of the first cue (t = -1 to 7 s). The data were downsampled to a sampling frequency of 300 Hz, after applying a notch filter to remove line noise and harmonics (at 50, 100, and 150 Hz). Bad channels and trials were rejected via visual inspection before independent component analysis (Jung et al., 2001) was applied. Subsequently, components representing eye-related and heart-related artifacts were projected out of the data (on average, three components were removed per participant). Finally, outlier trials of extreme variance were identified based on visual inspection and removed. This resulted in an average of 236 (± 3 SEM) trials and 266 channels per participant for the reported analyses.

Our analysis aimed to explore the impact of cueing and attentional modulation on oscillatory alpha dynamics using (1) univariate time–frequency analyses and (2) component-level analyses.

### 2.7 Univariate time–frequency analysis

We computed time–frequency representations of power for the full trials using an adaptive sliding time window of five cycles length per frequency (Δt = 5/f; 20-ms step size), and estimated power after applying a Hanning taper. We computed the planar representation of the MEG field distribution from the single-trial data using the nearest-neighbor method. This transformation facilitates the interpretation of the sensor level data as it makes the signal amplitude maximal above its source (as implemented in FieldTrip functions ft_megplanar and ft_combineplanar).

As a first step, we aimed to demonstrate global task-related modulations regardless of cueing or attentional modulation. This was done by contrasting oscillatory activity (4 to 35 Hz, and 8 to 13 Hz) between 0 and 6 s against baseline between -0.5 and -0.2 s. To control for aperiodic effects, we parametrized the baseline (-1 to 0 s) and task (0 to 6 s) spectral data with IRASA (Wen & Liu, 2016), in the frequency range of 2 to 40 Hz using default parameters as implemented in FieldTrip and extracted the slopes and intercepts. Afterward, we created (individualized) alpha-peak-centered, time-resolved signals with spectral bandwidths of 2 Hz for the cueing (pre- vs. retro-cue) and sample contrasts (attend first sample vs. attend second sample).

To create alpha-peak-centered signals, we extracted 1-s pre-stimulus windows (t = −1 to 0 s) for the 20 MEG channels displaying the maximal post-grating ERFs (channels selected individually per participant), multiplied these with a Hanning taper, and computed power spectra (1–30 Hz; 1-Hz resolution) using a fast Fourier transform (FFT) approach. We then detected the highest local maximum within the 7–14 Hz band to determine the individual alpha peak frequency (Haegens et al., 2014).

### 2.8 Source reconstruction

For each participant whose T1-weighted anatomical images were available (n = 13), an anatomically realistic single-shell headmodel based on the individual images was generated (Nolte, 2003). A grid with 0.5-cm resolution was created using an MNI template onto which the brain volume of those participants was morphed using non-linear transformation. For the remaining participants (n = 15), the template grid was used. For each grid point, leadfields were computed with a reduced rank, which removes the sensitivity to the direction perpendicular to the surface of the volume conduction model. This procedure ensures that each grid point represents the same anatomical location across all participants.

In order to visualize the sources of our sensor-level spectrotemporal effects, we used the frequency-domain adaptive spatial filtering technique of dynamical imaging of coherent sources (DICS; Gross et al., 2001). Data from all conditions of interest were concatenated in order to compute the cross-spectral density (CSD) matrices (t = -1 to 2 s; multitaper method (Mitra & Pesaran, 1999)) for the DICS beamformer. Leadfields for all grid points along with the CSD matrices were used to compute a common spatial filter (i.e., common for all trials and conditions) that was used to estimate the spatial distribution of power for our windows of interest. The source orientation was fixed to the dipole orientation with the highest strength.

### 2.9 Component-level analysis

For component-level analysis, we adopted the pipeline used by Rodriguez-Larios et al. (2022), except for our analysis not being restricted to posterior components. Similar to their pipeline, a series of conditions was imposed to select oscillatory components in the alpha range. First, independent components had to be classified as brain components by the IClabel algorithm (Pion-Tonachini et al., 2019) with a probability higher than 60%. Second, independent components had to show a maximum peak in the alpha range (as detected with the MATLAB *findpeaks* function). The frequency transformation of independent components was identical to the one used for time–frequency analysis described above. Single-trial alpha power was computed using the individual alpha band as defined per component based on its average power spectrum (across trials). This approach is meant to compensate for the lower signal-to-noise ratio of oscillatory activity when estimated in single trials.

To reconstruct the sources of the independent components, we used dipole fitting as implemented in FieldTrip after generating headmodels as described in the previous section. This resulted in x-, y-, and z-coordinates of the dipole location of each independent component.

### 2.10 Statistical analysis

In order to investigate the (sensor-level) differences between task-related activity and baseline, and the (sensor- and component-level) differences between the cueing conditions, we used nonparametric cluster-based permutation tests (Maris & Oostenveld, 2007). In brief, this test first calculates paired t-tests for each sensor at each time and/or frequency point, which are then thresholded at p < 0.05 and clustered on the basis of temporal, spatial, and/or spectral adjacency. The sum of t-values within each cluster is retained, and the procedure is repeated 1000 times on permuted data in which the condition assignment within each individual is randomized. On each permutation, the maximum sum is retained. Across all permutations, this yields a distribution of 1000 maximum cluster values. From this distribution, the probability of each empirically observed cluster statistic can be derived (evaluated at alpha = .05). To contrast the aperiodic parameters (slope and intercept), we used paired-sample t-tests.

For both the sensor-level univariate and the component-level approach, to assess the behavioral relevance of alpha power during the decision delay, that is, the 1-s time window before the onset of the response mapping, we computed single-trial alpha power using the individual alpha band (peak frequency +/-2 Hz) as defined per participant based on the average power spectrum (across all trials). We split the trials based on alpha power (median split) and used a t-test to contrast accuracy and reaction time between high and low alpha power trials.

Next, to investigate the impact of our attentional and cueing manipulations on alpha power, we focused our analysis on the 1-s time window preceding the sample onset. We computed single-trial alpha power in the same way described above, pooled the data preceding the two samples, and labeled them as either attended or unattended. Note that in the retro-cue condition, the participant only learns which sample is to be attended *after* sample presentation. To assess the effect of condition on pre-stimulus alpha power (at both sensor and component levels), we conducted a 2 x 2 ANOVA with factors cueing (pre vs. retro) and relevance (attended vs. unattended).

## 3 Results

### 3.1 Behavioral results

We recorded MEG data from 28 healthy participants who performed a delayed match-to-sample working-memory paradigm that involved comparing the spatial frequency of one of two visual grating samples with that of a probe. The to-be-matched grating was indicated by a visual cue occurring either before (pre-cue) or after (retro-cue) sample presentation.

Overall, participants were faster in the retro- than in the pre-cue condition (Fig. 1B; F(1,27) = 15.5, p = 0.0001). There was no significant difference in RT between relevant sample conditions (first vs. second sample; F(1,27) = 1.9, p = 0.16), nor a significant difference between probe onset conditions (early, late, or jittered; F(2,27) = 1.5, p = 0.21). In terms of accuracy, participants performed better in the retro-cue condition (Fig. 1C; F(1,27) = 12.2, p = 0.0005). There was a significant modulation of accuracy by probe onset (Fig. 1D; F(2,27) = 4.8, p = 0.008), which was mainly driven by better performance when the probe arrived at an early compared with late onset (early vs. late; t(27) = 3.1, p = 0.007). There was no significant difference in accuracy between sample conditions (F(1,27) = 2.8, p = 0.1).

### 3.2 Univariate analysis shows task-related alpha modulation

**Fig. 2.**
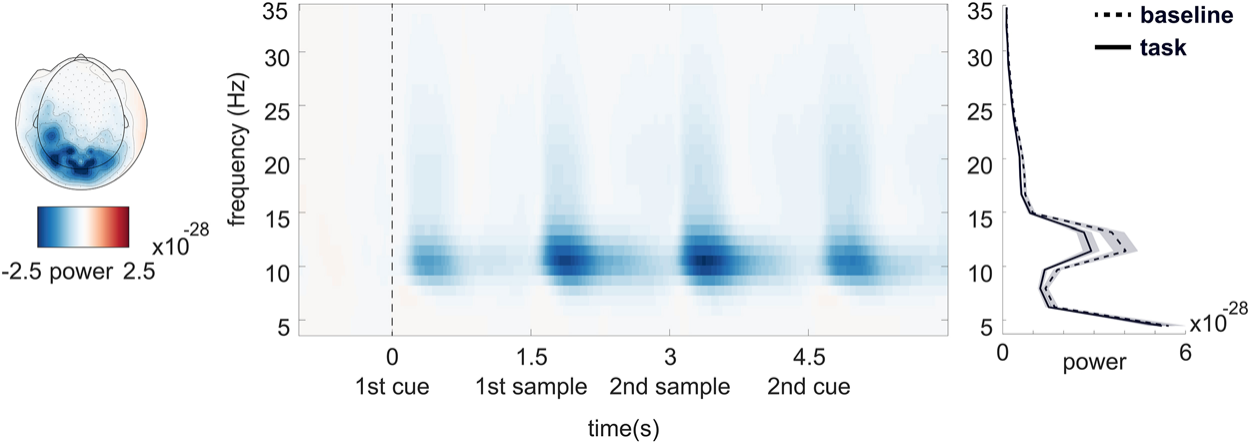
Task-related alpha power modulation. Time–frequency representation (middle) and power spectrum (right) of oscillatory power during the working-memory task compared with baseline activity. Topography (left) shows the distribution of the alpha-band modulation in sensor space.

We first investigated task-related modulations by contrasting oscillatory activity (4 to 35 Hz) during the task (0 to 6 s) against baseline activity (-0.5 to -0.2 s) using mass univariate analysis. We found a significant decrease in oscillatory power following cue onset (Fig. 2; p_cluster_ < 0.001; time = 0.15 to 6 s; frequency = 6 to 35 Hz). The power decrease spanned both alpha and beta bands; however, since the focus of the current study is alpha, we additionally tested the alpha band separately (Fig. 2; p_cluster_ < 0.001; time = 0.15 to 6 s; frequency = 8 to 13 Hz). To make sure this reflected a genuine oscillatory alpha modulation, and not an aperiodic 1/f effect, we parametrized the baseline vs. task spectra with IRASA and found that neither the slope nor intercept was statistically different. In summary, alpha activity was modulated during the task as compared with baseline, and even though beta activity was also modulated, these changes were not due to aperiodic 1/f effects.

To assess the behavioral relevance of alpha power modulations, we compared accuracy and reaction time between trials with high and low alpha power (median split) during the decision window. We found no significant differences in accuracy (t(27) = 0.2347, p = 0.8162) or reaction times (t(27) = -1.1018, p = 0.2803) between trials with high vs. low univariate alpha power (Supplementary Fig. S1).

### 3.3 Component analysis reveals distinct alpha sources associated with task performance

**Fig. 3.**
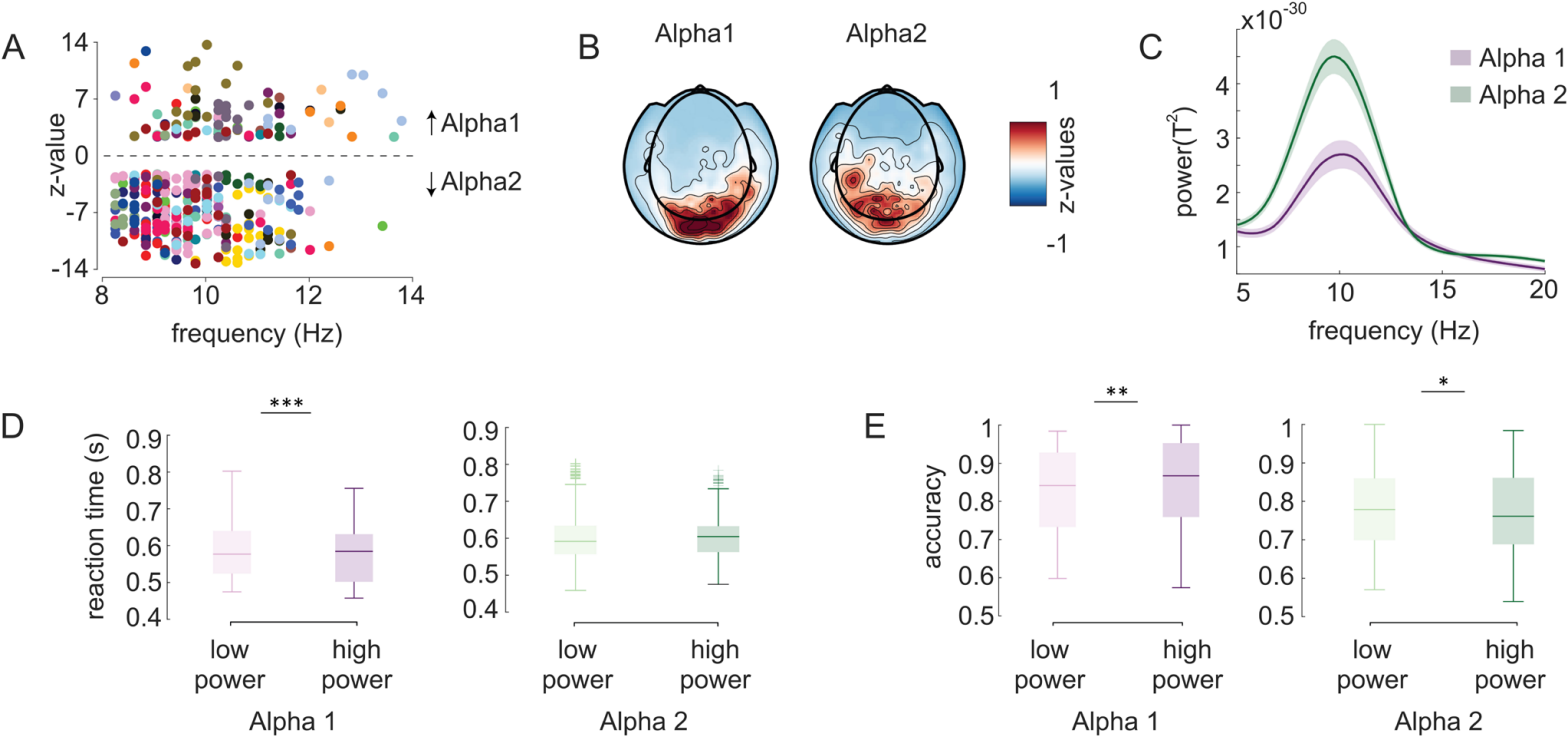
Alpha components during decision window. (A) Power changes per component during the decision delay window relative to baseline. Z-values plotted as a function of components’ peak frequencies (each color codes for a different subject). Components showing a significant increase in power during the decision delay were denominated Alpha1, while components showing a significant decrease were denominated Alpha2. (B) Mean topographies of the power change for Alpha1 (left) and Alpha2 components (right). (C) Average spectra of Alpha1 (purple) and Alpha2 components (green) during the decision delay (shaded area depicts standard error of the mean). (D) Effect of power modulation on reaction time, comparing high and low power trials (median split) per component type. Participants were faster on trials where Alpha1 components increased in power. (E) Effect of power modulation on accuracy. Participants were more accurate on trials where Alpha1 components increased in power and on trials where Alpha2 components decreased in power. Within each boxplot, the horizontal line represents the median, the box delineates the area between the first and third quartiles (interquartile range). *p < 0.05, **p < 0.01, ***p < 0.001.

We then turned to component-level analysis. Aiming to replicate and extend the findings by Rodriguez-Larios et al. (2022) whose analysis focused on a working-memory delay, we first focused our ICA decomposition on the decision window (i.e., 1 s prior to response mapping presentation). Indeed, we found distinct clusters of alpha components with two distinct profiles (Fig. 3A, B, C): 95 alpha components whose power increased during the decision window compared with baseline (Alpha1), and 285 alpha components whose power decreased (Alpha2). The breakdown across participants was as follows: 20 participants showed both types of alpha components, 2 participants showed only Alpha1 components, 5 participants showed only Alpha2 components, and 1 participant showed no significant alpha components.

Contrary to the sensor-level analysis, we found that for Alpha1 components, trials with high power during the decision window were associated with better performance, that is, faster reaction times (Fig. 3D left panel; t(94) = -4.69, p_corr_ = 0.0001) and higher accuracy (Fig. 3E left panel; t(94) = 3.42, p_corr_ = 0.001), than trials with low power. For Alpha2 components, we found the opposite relationship between power and accuracy, that is, trials with low power were associated with higher accuracy (Fig. 3E right panel; t(284) = -2.52, p_corr_ = 0.024), though the difference in reaction time was not significant (Fig. 3D right panel; t(284) = 2.22, p_corr_ = 0.054) despite trending in the expected direction.

In summary, we extended previous findings demonstrating the existence of at least two distinct sources (Alpha1 and Alpha2) with opposite power modulations during a working-memory task, and showed a similar effect during the decision window. Additionally, we found that better task performance was associated with higher Alpha1 power and lower Alpha2 power (while no performance differences were observed in the univariate analysis). In other words, for the alpha component that increased in power during the decision delay, a further increase was beneficial for behavior, and for the alpha component that decreased in power, a further decrease was beneficial for behavior.

### 3.4 Component analysis reveals impact of cueing on task-related alpha modulation

**Fig. 4.**
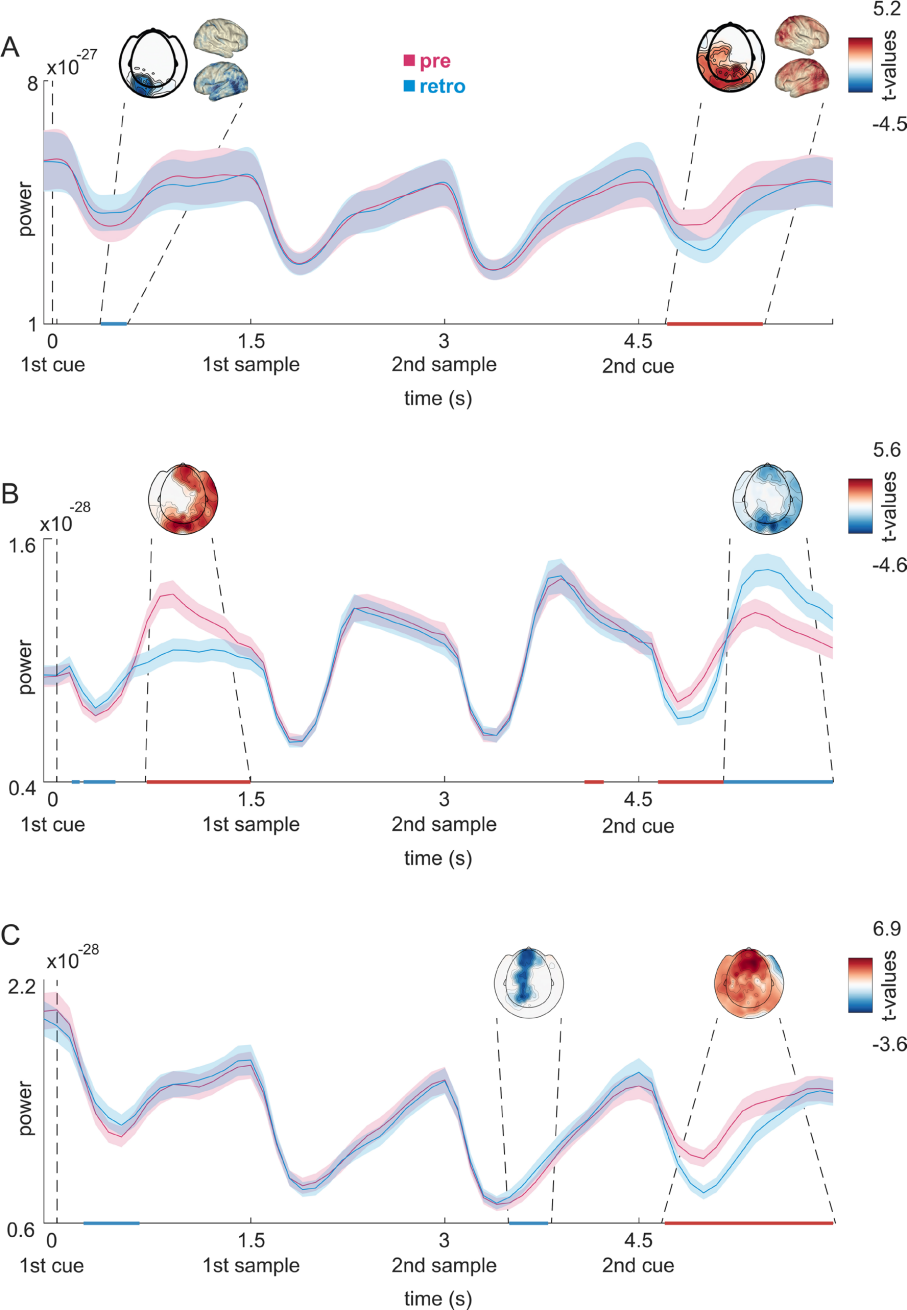
Cue-related alpha power modulation. (A) Cue-related alpha time-courses for the pre- (pink) and retro-cue (blue) conditions on sensor level. Shaded areas denote between-participants standard error, and red (pre>retro) and blue (pre<retro) bars indicate time points with significant differences between conditions (p < 0.05). Topographies show statistical distributions of these significant differences. Brain plots represent the topographies projected to source space. (B) Similar to A for component-level analysis, showing Alpha1 components and (C) Alpha2 components.

We then investigated the impact of cueing on alpha power, by contrasting alpha-centered time-courses of oscillatory power between pre- and retro-cue conditions, starting with the sensor-level alpha time-courses. We found a similar cueing effect as in our previous study (ElShafei et al., 2022). Following the first cue, alpha decrease was stronger for the pre-cue condition (in which the first cue was informative) in comparison with the retro-cue condition (in which the first cue was uninformative; Figure 4A; p_cluster_ = .008; time = 0.34 to 0.54 s; effect centered on occipital sensors). These differences between pre- and retro-cue conditions following first cue presentation were mainly localized to left and right occipital cortices (Fig. 4A insets). The pattern was reversed following the second cue, with alpha power decrease being stronger for the retro-cue condition (in which the second cue was informative) than in the pre-cue condition (in which the second cue was uninformative; Fig. 4A; p_cluster_<.001; time = 4.7 to 5.5 s; effect centered on occipitoparietal and temporal sensors). These differences between pre- and retro-cue conditions following second cue presentation were mainly localized to left and right occipital cortices and left middle and superior temporal gyri (Fig. 4A insets).

For the Alpha1 components (Fig. 4B), the most prominent cue-related effects were an increase in Alpha1 power in the pre- vs. retro-cue condition following the presentation of the first cue (0.6 to 1.5 s; p_cluster_<.001), and a decrease following the second cue (5.2 to 6 s; p_cluster_<.001). Other significant effects included increases in Alpha1 power following the second cue (4.6 to 5.2 s; p_cluster_<.001) and following the second sample (4.1 to 4.2 s; p_cluster_ = .025), and decreases in Alpha1 power following the first cue (0.2 s; p_cluster_ = .024; and 0.3 to 0.6 s; p_cluster_ = .01).

For the Alpha2 components (Fig. 4C), the most prominent cue-related effect was an increase in Alpha2 power in the pre- vs. retro-cue condition following the presentation of the second cue (4.6 to 6 s; p_cluster_ < 0.001). Other significant effects included decreases in Alpha2 power following the second sample (3.4 to 3.9 s; p_cluster_ = .006) and following the first cue (0.3 to 0.7 s; p_cluster_ = .017).

In sum, our sensor-level, univariate time–frequency analysis showed that alpha power decreased following cue presentation, and more so following an informative (vs. uninformative) cue. These modulations were more pronounced on the component level, with the main Alpha2 effect consistent with that observed on sensor level, while Alpha1 additionally showed opposite dynamics. It appears that on sensor level, these Alpha1 dynamics may have been masked by Alpha2 activity, which overall emphasizes the limitations of univariate analysis.

### 3.5 Component analysis reveals impact of attention conditions on pre-stimulus alpha

**Fig. 5.**
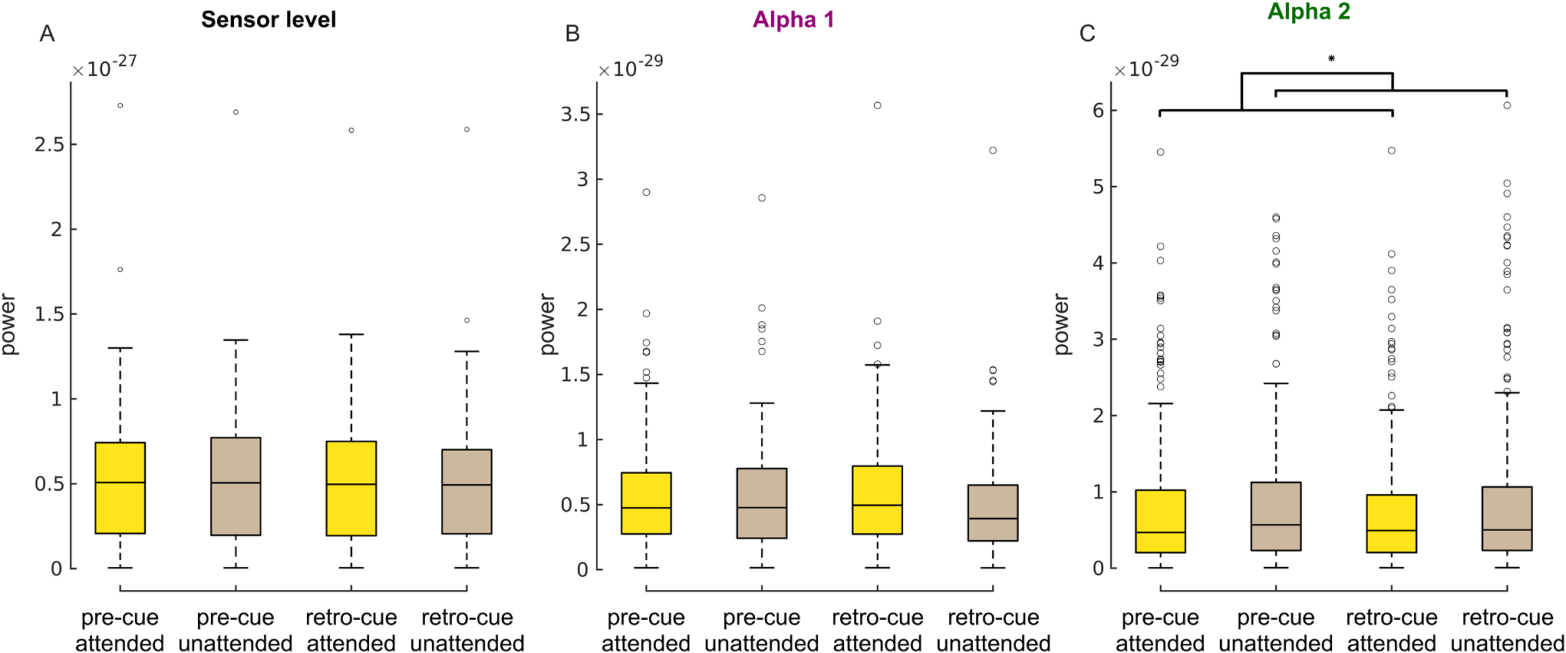
Pre-stimulus alpha power modulation by task condition. (A) Pre-stimulus alpha power at sensor level, per task condition (cue x relevance). (B) Same as A for component-level analysis, showing Alpha1 and (C) Alpha2. Within each boxplot, the horizontal line represents the median, the box delineates the area between the first and third quartiles (interquartile range). *p < 0.05.

Next, we investigated the impact of our attentional manipulations on anticipatory alpha power, at both sensor and component levels. For this purpose, we pooled all samples (i.e., first and second, all conditions) and focused our analysis on the 1-s time window preceding sample onset (i.e., the pre-stimulus window). We used a 2 x 2 ANOVA with the factors cueing (pre vs. retro) and relevance (attended vs. unattended). At sensor level, there were no main effects of cueing and relevance on pre-stimulus alpha power (Fig. 5A; F(1,27 = 0.07, p = 0.79; F(1,27) = 0.04, p = 0.83, respectively). To further investigate potential modulations at the component level, we analyzed Alpha1 and Alpha2 power prior to sample presentation using the same factorial design. For Alpha1, neither cueing nor relevance had a significant effect on pre-stimulus power (Fig. 5B; F(1,94) = 0.74, p = 0.39; F(1,94) = 0.70, p = 0.40, respectively). In contrast, for Alpha2, there was a significant main effect of relevance with greater pre-stimulus power observed for unattended than for attended samples (Fig. 5C; F(1,284) = 4.86, p = 0.02). There was no significant main effect of cueing (F(1,284) = 0.01, p = 0.90), and no significant interaction between cueing and relevance (F(1,284) = 0.34, p = 0.56).

Taken together, these analyses suggest that while sensor-level data did not reveal cue x relevance effects on pre-stimulus alpha power, the component analyses demonstrated increased Alpha2 power prior to the unattended compared with attended sample.

### 3.6 Component analysis reveals distinct relationships between pre-stimulus alpha power and task performance

**Fig. 6.**
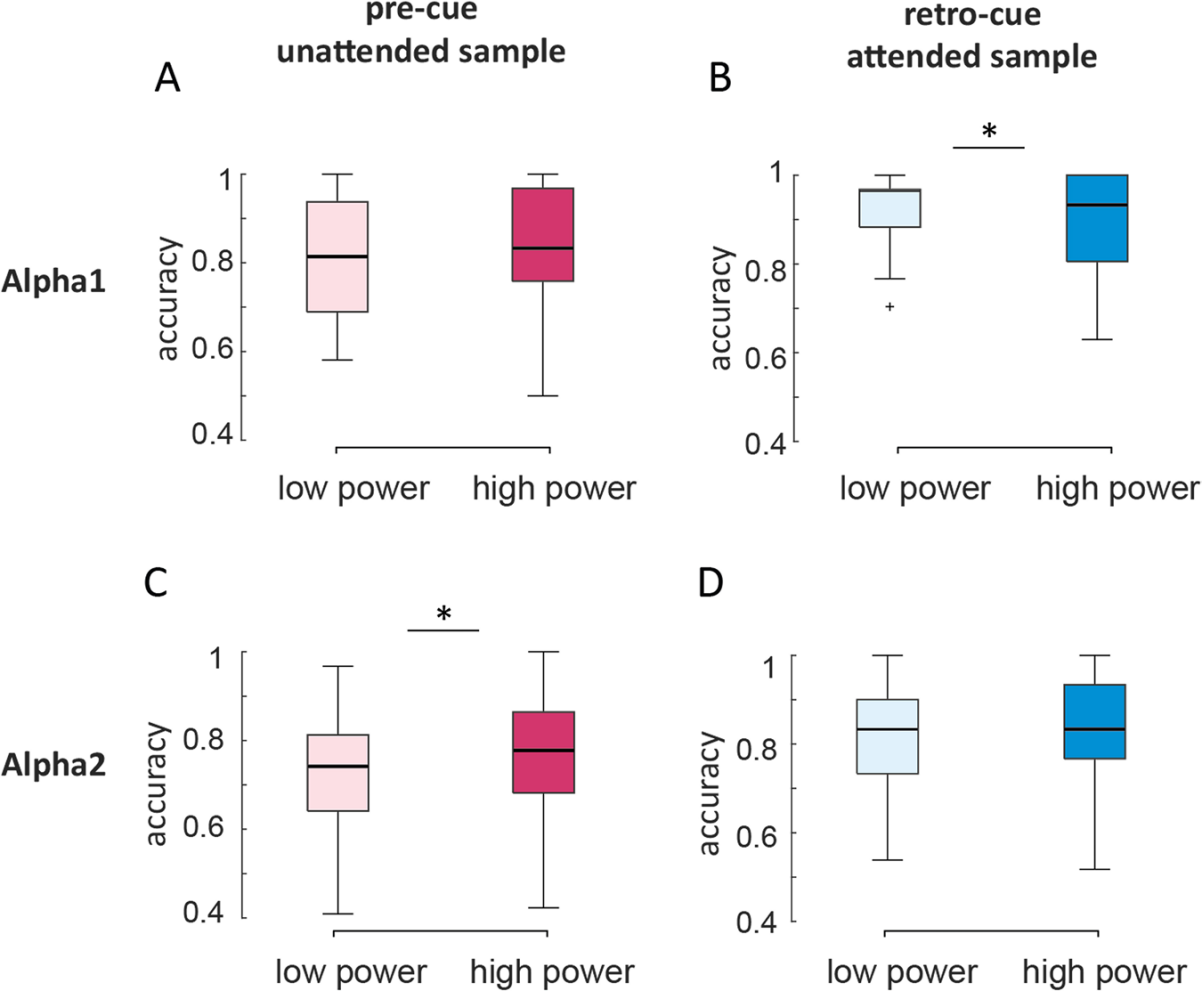
Relationship between component-level pre-stimulus alpha power and performance per task condition. (A) Effect of pre-stimulus power modulation on accuracy for the unattended sample in the pre-cue condition. Accuracy was compared between low- and high-power trials (median split) for Alpha1 components, showing no significant difference. (B) Same as A for the attended sample in the retro-cue condition, showing significantly higher accuracy for trials with low Alpha1 power prior to sample presentation. (C) Same as A for Alpha2 components, showing significantly higher accuracy for trials with high Alpha2 power prior to sample presentation. (D) Same as B for Alpha2 components, showing no significant difference. Within each boxplot, the horizontal line represents the median, the box delineates the area between the first and third quartiles (interquartile range). *p_corr_ < 0.05.

We then investigated the impact of anticipatory alpha power modulations on behavioral performance, as a function of our attentional manipulations (Fig. 6). We compared accuracy and reaction time between trials with high and low pre-stimulus alpha power (median split) considering cueing (pre- vs. retro-cue) and attention (attended vs. unattended) conditions. Note that in the retro-cue condition, the participant only learns which sample is to be attended after sample presentation.

For the sensor-level analysis, we found no significant difference in accuracy in any of the contrasts (Supplementary Fig. S2): pre-cue attended (t(27) = 2.43, p_corr_ = 0.088), pre-cue unattended (t(27) = 0.93, p_corr_ = 1), retro-cue attended (t(27) = 1.61, p_corr_ = 0.472), and retro-cue unattended (t(27) = -0.22, p_corr_ = 1). Additionally, we found no significant difference in reaction times in any of the contrasts (Supplementary Fig. S3): pre-cue attended (t(27) = -0.18, p_corr_ = 1), pre-cue unattended (t(27) = 0.48, p_corr_ = 1), retro-cue attended (t(27) = -0.94, p_corr_ = 1), and retro-cue unattended (t(27) = -0.34, p_corr_ = 1).

For the component-level analysis, we found a negative relationship between pre-stimulus Alpha1 power and accuracy for the attended samples in the retro-cue condition (Fig. 6B; t(94) = -2.5, p_corr_ = 0.016), but no significant accuracy differences for Alpha1 power in the other contrasts (Fig. 6A and Supplementary Fig. S4): pre-cue attended (t(94) = 0.02, p_corr_ = 0.982), pre-cue unattended (t(94) = 1.1, p_corr_ = 0.254), and retro-cue unattended (t(94) = -1.6, p_corr_ = 0.124). In other words, in trials where participants did not know which sample to attend to, higher Alpha1 power prior to the to-be-attended stimulus led to worse performance.

In addition, we found a positive relationship between pre-stimulus Alpha2 power and accuracy for the unattended samples in the pre-cue condition (Fig. 6C; t(284) = 3, p_corr_ = 0.003), but no significant accuracy differences for Alpha2 power in the other contrasts (Fig. 6D and Supplementary Fig. S4): pre-cue attended (t(284) = 1.8, p_corr_ = 0.068), retro-cue attended (t(284) = 0.6, p_corr_ = 0.498), and retro-cue unattended (t(284) = 1.3, p_corr_ = 0.196). In other words, in trials where participants knew which sample to attend to, higher Alpha2 power prior to the irrelevant sample led to better performance.

Finally, we found no significant differences in reaction times for Alpha1 and Alpha2 in any of the contrasts (Supplementary Figs. S5 and S6): pre-cue attended (Alpha1, t(94)= -1.4, p_corr_ = 0.148; Alpha2, t(284) = 0.4, p_corr_ = 0.656), pre-cue unattended (Alpha1, t(94) = -1.8; p_corr_ = 0.072; Alpha2, t(284) = 1.6, p_corr_ = 0. 108), retro-cue attended (Alpha1, t(94) = -1.0, p_corr_ = 0.321; Alpha2, t(284) = -0.09, p_corr_ = 0.9), and retro-cue unattended (Alpha1, t(94) = -0.8, p_corr_ = 0.432; Alpha2, t(284) = -0.6, p_corr_ = 0.541).

In summary, we demonstrated distinct relationships between pre-stimulus Alpha1 and Alpha2 power and task performance, dependent on cueing and attention conditions. Alpha1 power was negatively associated with accuracy when the task relevance of to-be-attended input was unknown, whereas Alpha2 power was positively associated with accuracy when the relevance of to-be-unattended input was known. No such effects were observed in the sensor-level analysis.

### 3.7 The differential spatiospectral characteristics of Alpha1 and Alpha2 components

**Fig. 7.**
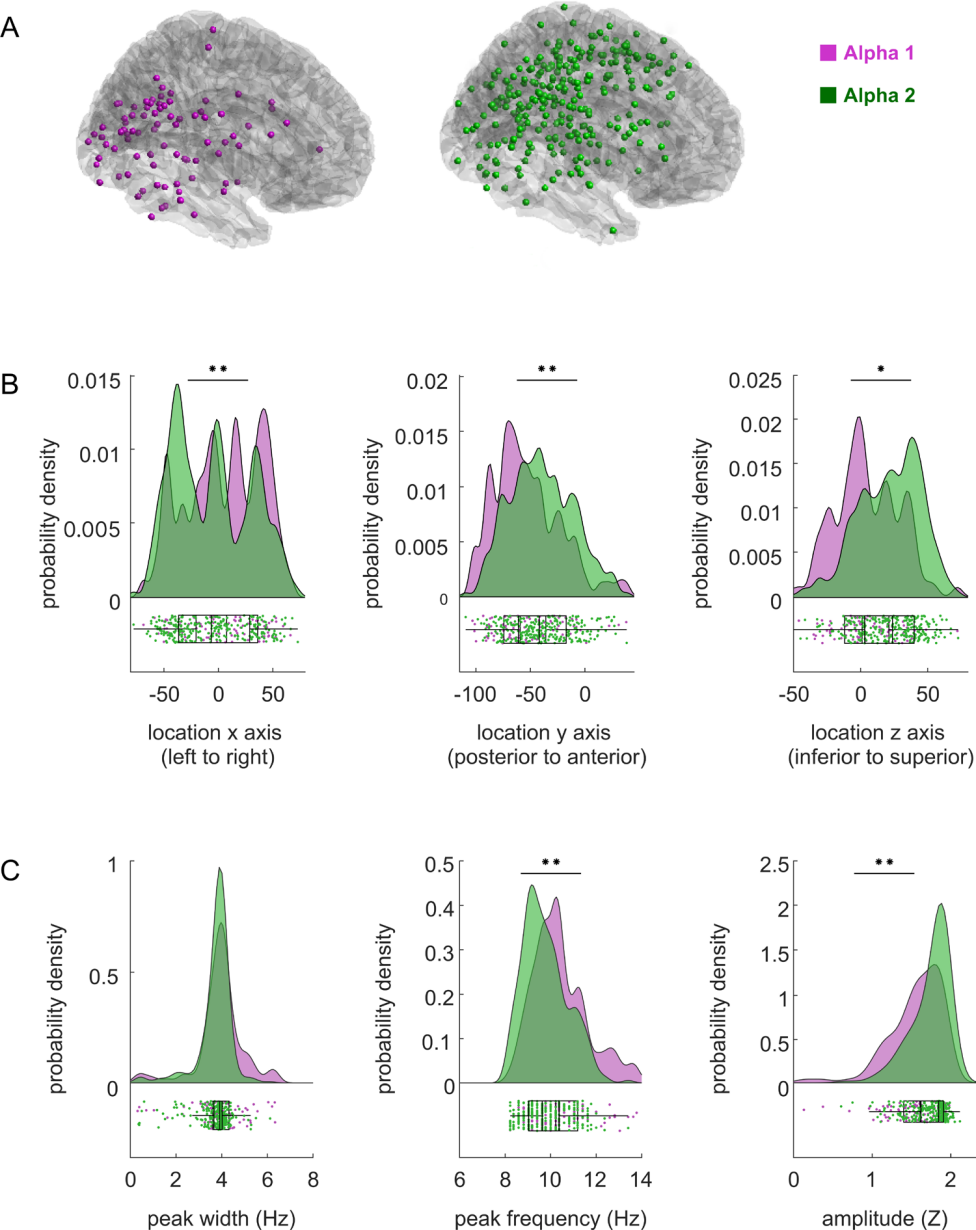
Spatiospectral differences between Alpha1 and Alpha2 components. (A) Source localization of Alpha1 (purple) and Alpha2 (green) components as estimated with dipole fitting. (B) Location of Alpha1 and Alpha2 components in source space on x-axis (left to right; left panel), y-axis (posterior to anterior; middle), and z-axis (inferior to superior; right). (C) Spectral parameters for Alpha1 and Alpa2 components: peak width (left), frequency (middle), and amplitude (right). *p_corr_ < 0.016, **p_corr_ < 0.001.

Finally, to investigate whether Alpha1 and Alpha2 differ in their spatiospectral profiles, we compared three spectral parameters (peak frequency, peak width, and amplitude), and the location of the estimated main sources using dipole fitting (Fig. 7A). We found that Alpha1 components tended to have more right-hemispheric (t(362) = -5.64, p_corr_ < 0.001), posterior (t(362) = 2.44, p_corr_ < 0.016), and inferior (t(362) = -3.56, p_corr_ < 0.001) source localization than Alpha2 components (Fig. 7B). We observed no difference in peak width (t(377) = 1.45, p_corr_ = 0.4403), while Alpha1 showed a higher peak frequency than Alpha2 (t(377) = 5.19, P_corr_ < 0.001), and Alpha2 exhibited a stronger relative amplitude than Alpha1 t(377) = -4.99, p_corr_ < 0.001; Fig. 7C).

## 4 Discussion

We used a visual working-memory task with pre- and retro-cueing to investigate the role of alpha oscillations in gating information processing, and the impact of top-down attention on these dynamics. Consistent with the proposal that alpha regulates information flow by inhibiting task-irrelevant and disinhibiting task-relevant processing (Jensen & Mazaheri, 2010; Klimesch, 2012; Klimesch et al., 2007), we observed an alpha power decrease that temporally tracked the presentation of the informative cue. This observation of stronger alpha power decrease following the informative cue replicates our recent work (ElShafei et al., 2022). Furthermore, using an ICA approach allowed detection of two distinct clusters of alpha components with opposite power modulations during the decision delay, which were differentially associated with task performance. Additionally, our decomposition approach identified how pre-stimulus power in these two distinct alpha sources differentially affected task performance dependent on task relevance of the sample stimulus. Importantly, univariate sensor-level analysis did not reveal any correlations with task performance.

Note that unlike ElShafei et al., we observed higher accuracy and faster responses in the retro-cue condition. This retro-cue benefit may stem from the high representational similarity between samples, with retrospective cues likely reducing the interference in working memory (Griffin & Nobre, 2003; Pertzov et al., 2013; Souza & Oberauer, 2016).

The existence of multiple alpha sources with functional implications dependent on their spatial origin and the task context aligns with established theories of alpha (Jensen & Mazaheri, 2010; Klimesch et al., 2007). Previous studies suggested spatially distinct alpha sources differentially mediate attention and visual perception (Gulbinaite et al., 2017; Sokoliuk et al., 2019). Notably, recent work revealed the simultaneous existence of multiple posterior alpha sources with opposite power modulation during working-memory maintenance, which traditional sensor-level analysis could not identify (Rodriguez-Larios et al., 2022). Rodriguez-Larios et al. argued that Alpha1 increase during memory retention reflects the inhibition of lower-order areas involved in visual processing (i.e., to block interfering input; Bonnefond & Jensen, 2013; Jensen, 2002; Tuladhar et al., 2007), and Alpha2 decrease reflects the disinhibition of higher-order areas involved in transient storage of visual information (De Vries et al., 2020; Van Ede, Jensen, et al., 2017). Accordingly, they showed that behavioral performance improves with power increases in Alpha1 and with decreases in Alpha2 during memory retention. Here we applied the same ICA approach to the decision delay, and similarly identified two distinct alpha sources with opposite power modulations. Crucially, we extend the previous findings by showing that better performance was associated with a further increase of the component that increased in power (Alpha1) and a further decrease in the component that decreased in power (Alpha2) during the decision delay.

Investigating pre-stimulus alpha modulation as a function of our cueing conditions, we found that the modulation of alpha sources and its subsequent impact on task performance varied based on cue informativeness and whether the upcoming input was task relevant. When task relevance was unknown (i.e., in the retro-cue condition), increases in Alpha1 power before the task-relevant input were associated with poorer performance. In contrast, when task relevance was known (i.e., in the pre-cue condition), pre-stimulus increases in Alpha2 power before the task-irrelevant (i.e., to-be unattended) input were associated with better task performance. Anticipatory alpha power increases related to unattended input have previously been found to positively correlate with behavioral performance, and are typically interpreted as reflecting the inhibition of areas involved in processing task-irrelevant information that could potentially interfere with task performance (Bonnefond & Jensen, 2012; Haegens et al., 2012; Payne et al., 2013; Mazaheri et al., 2014). Conversely, alpha increases in areas processing task-relevant input tend to negatively correlate with performance (Haegens et al., 2012; Iemi et al., 2022; Zhou et al., 2021). Our findings of a negative association of performance with pre-stimulus Alpha1 power, and a positive association with Alpha2 power, are in line with this interpretation. In this view, increases in pre-stimulus Alpha1 power impaired retention of the relevant information, possibly via the inhibition of networks involved in visual processing, while increases in Alpha2 power impaired processing of the irrelevant information, possibly via the inhibition of networks involved in transient storage of visual information.

Previous studies on distractor suppression-related alpha activity have reported inconsistent results, as some found alpha power increases before task-irrelevant information (Bonnefond & Jensen, 2012; Haegens et al., 2012; Payne et al., 2013; Snyder & Foxe, 2010; Wang et al., 2019), while others did not (Noonan et al., 2016; Van Moorselaar & Slagter, 2019). Pre-stimulus alpha activity as observed on sensor level reflects a mixture of oscillatory sources with potentially different functionality and neurophysiological characteristics (Benwell et al., 2019; Palva & Palva, 2012). Only a few studies have spatially separated alpha sources or designed their experiments to distinguish neuronal sources processing the relevant vs. irrelevant information (Bonnefond & Jensen, 2024). Here, using a decomposition approach, we show attentional differences that were not detected using univariate analysis. Given our current observations and those reported by Rodriguez-Larios et al. (2022), conflicting reports in the literature may be explained by the coexistence of multiple alpha sources with opposing power modulations in certain task contexts. These opposing modulations might cancel each other out when observed with traditional univariate methods. While we acknowledge that differences in experimental design might further contribute to inconsistent alpha findings (Bonnefond & Jensen, 2024; Jensen, 2024; Noonan et al., 2018), we suggest that future studies consider decomposition or other source separation methods to dissociate different alpha components and their contribution to anticipatory attention.

In conclusion, we report on preparatory mechanisms involving two distinct alpha sources, whose power modulations before stimulus presentation and during the decision delay are differentially related to behavioral outcomes. We propose that increases in Alpha1 power reflect the inhibition of visual processing, and accordingly show that increase in Alpha1 prior to task-relevant stimulus presentation leads to poorer task performance, while increase during the decision delay (i.e., when visual input would interfere) leads to better task performance. Additionally, we propose that increases in Alpha2 power reflect the inhibition of transient storage of visual information, and accordingly show that both increase in Alpha2 power prior to processing of task-irrelevant information and decrease of Alpha2 during the decision delay (i.e., when maintenance of the decision is required) are associated with better performance. In other words, the alpha component whose increase is beneficial during the decision delay is detrimental prior to a task-relevant stimulus, and vice-versa. Notably, these distinct alpha dynamics and the attentional modulation of anticipatory alpha became evident only after using ICA, highlighting the necessity of analysis methods that can disentangle overlapping rhythmic dynamics that stem from different sources.

## Supplementary Material

Supplementary Material

## Data Availability

Data and code are available here: https://osf.io/gkbsm/
