# Supplementary material for: Functionally distinct alpha components are differentially modulated by attention and affect behavior
Source: Imaging Neurosci (Camb). 2026 Aug 17;4:IMAG.a.1335. doi: 10.1162/IMAG.a.1335 (PMC13483082; doi:10.1162/IMAG.a.1335)
Supplement: Supplementary file 1 [file IMAG.a.1335_supp.pdf]

Supplementary Materials

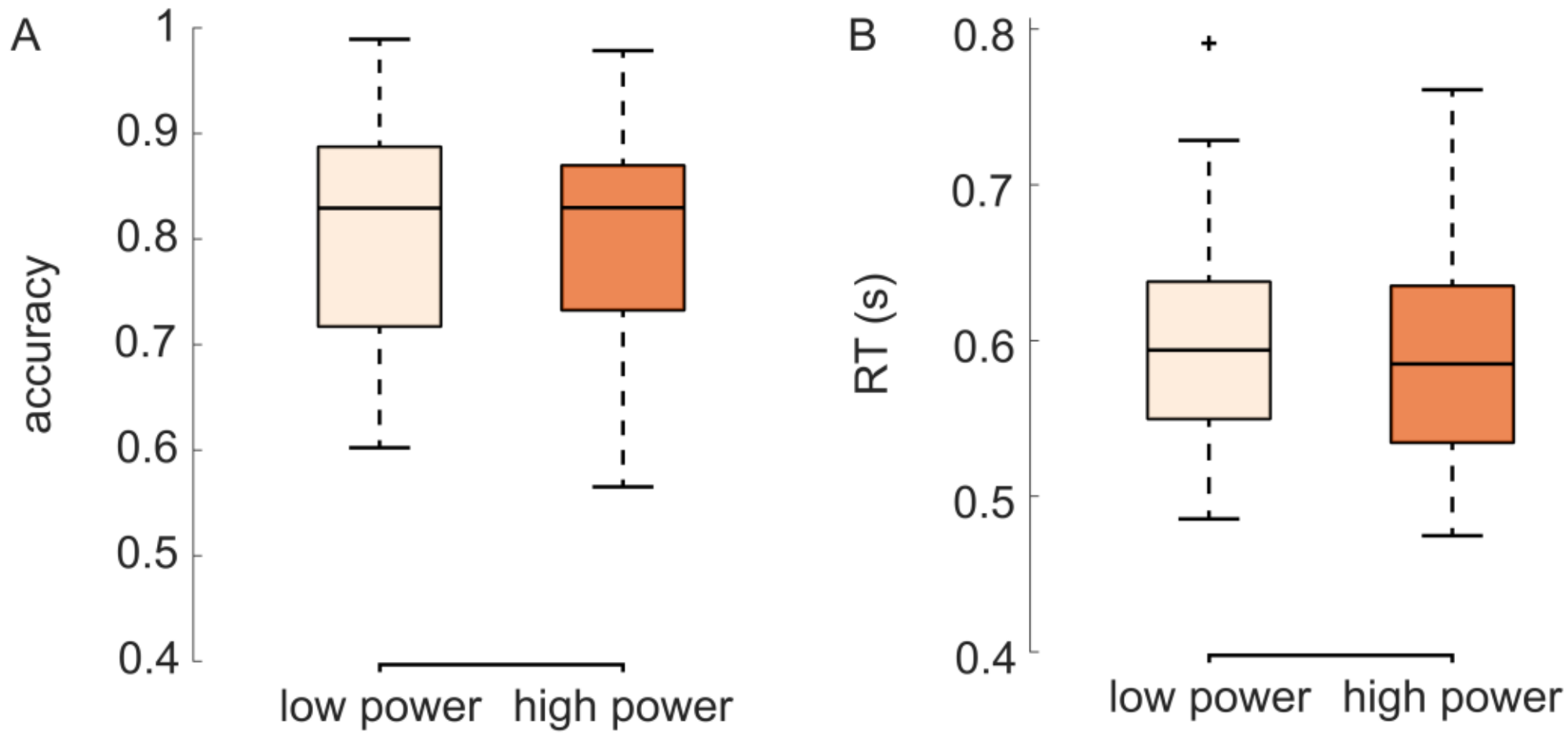

Figure S1. Relationship between sensor-level alpha power during the decision window and performance.

[A] Effect of alpha power modulation on accuracy during the decision delay. Accuracy was compared between low- and high-power trials (median split), showing no significant difference. [B] Same as A, but showing the effect of power modulation on reaction times (RT). Within each boxplot, the horizontal line represents the median, the box delineates the area between the first and third quartiles (interquartile range).

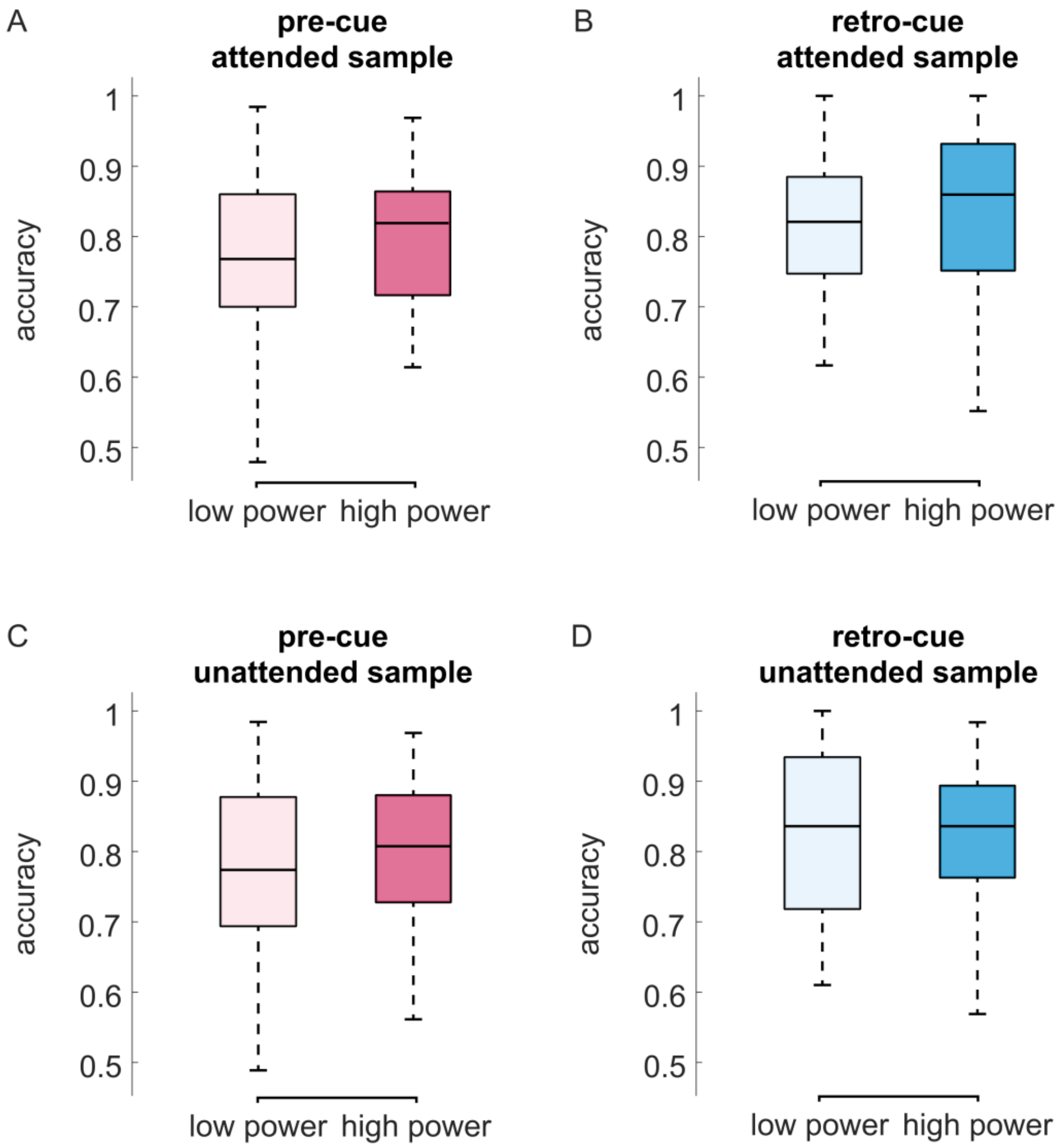

Figure S2. Relationship between sensor-level pre-stimulus alpha power and accuracy per task condition.

[A] Effect of pre-stimulus alpha power modulation on accuracy for the attended sample in the pre-cue condition. Accuracy was compared between low- and high-power trials (median split), showing no significant difference. [B] Same as A for the attended sample in the retro-cue condition. [C] Same as A for the unattended sample in the pre-cue condition. [D] Same as A for the unattended sample in the retro-cue condition. Within each boxplot, the horizontal line represents the median, the box delineates the area between the first and third quartiles (interquartile range).

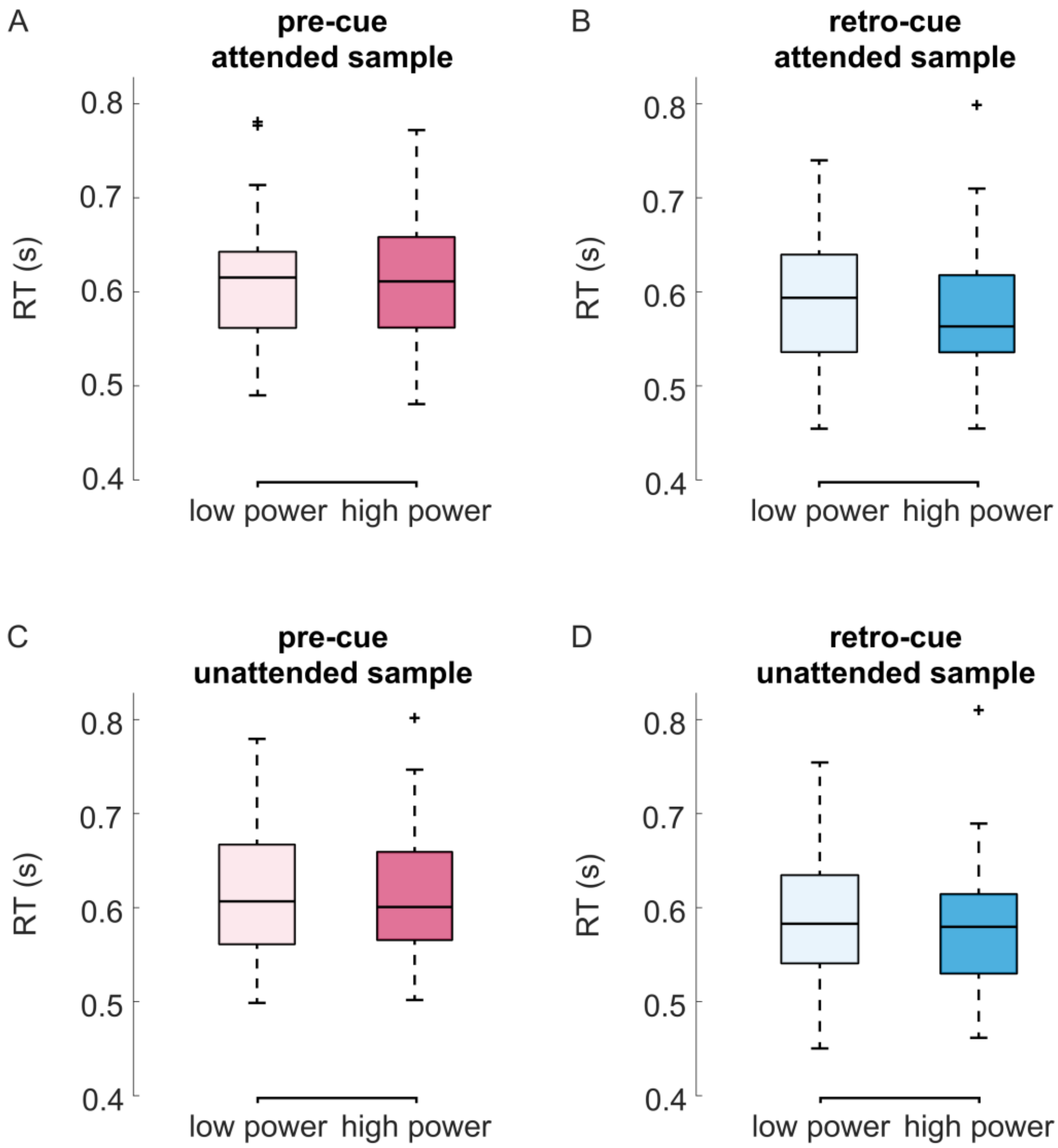

Figure S3. Relationship between sensor-level pre-stimulus alpha power and RT per task condition.

[A] Effect of pre-stimulus alpha power modulation on reaction times (RT) for the attended sample in the pre-cue condition. RT was compared between low- and high-power trials (median split), showing no significant difference. [B] Same as A for the attended sample in the retro-cue condition. [C] Same as A for the unattended sample in the pre-cue condition. [D] Same as A for the unattended sample in the retro-cue condition. Within each boxplot, the horizontal line represents the median, the box delineates the area between the first and third quartiles (interquartile range).

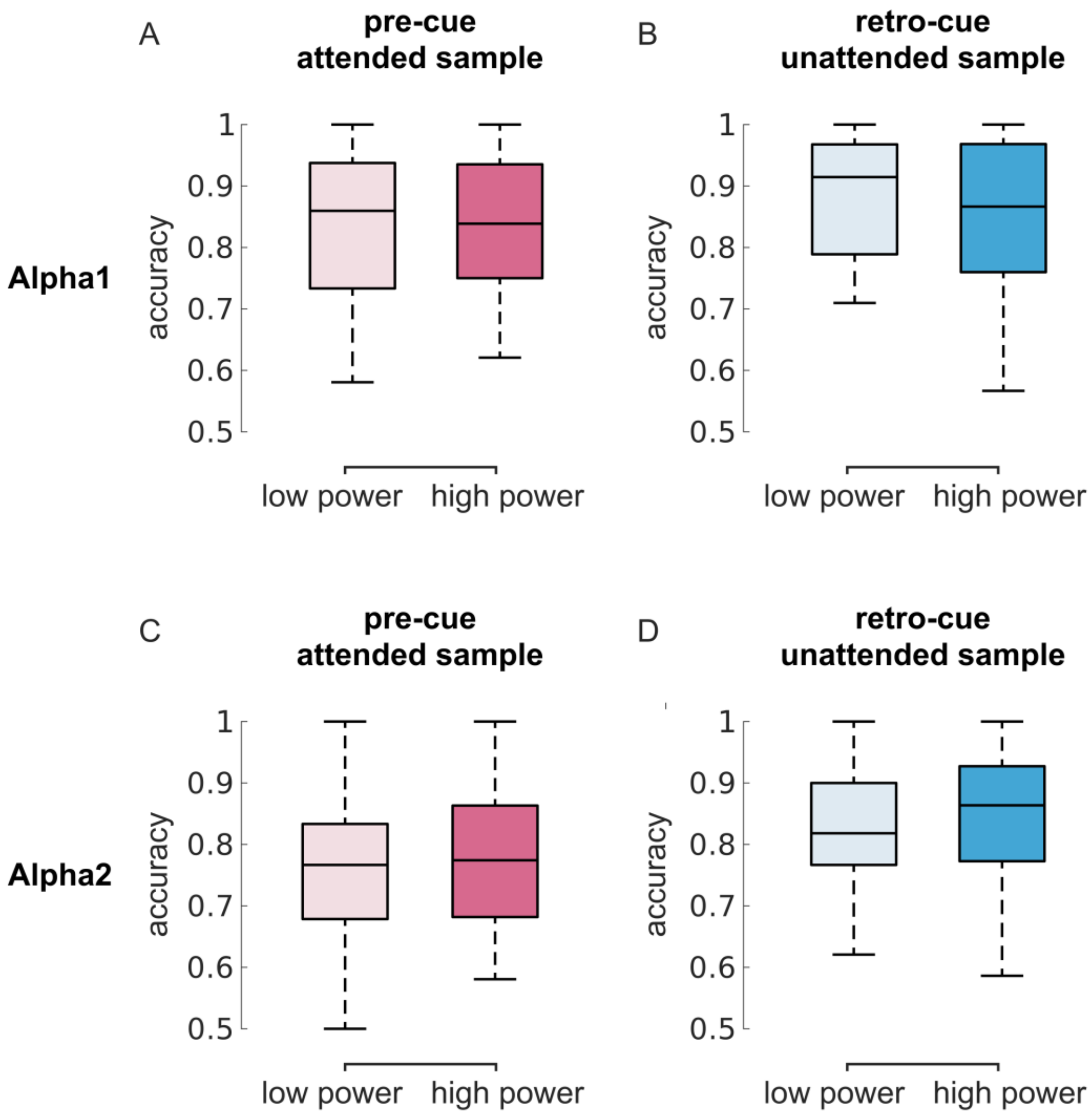

Figure S4. Relationship between component-level pre-stimulus alpha power and performance per task condition.

[A] Effect of pre-stimulus power modulation on accuracy for the attended sample in the pre-cue condition. Accuracy was compared between low- and high-power trials (median split) for Alpha1 components, showing no significant difference. [B] Same as A for the unattended sample in the retro-cue condition. [C] Same as A for Alpha2 components. [D] Same as B for Alpha2 components. Within each boxplot, the horizontal line represents the median, the box delineates the area between the first and third quartiles (interquartile range).

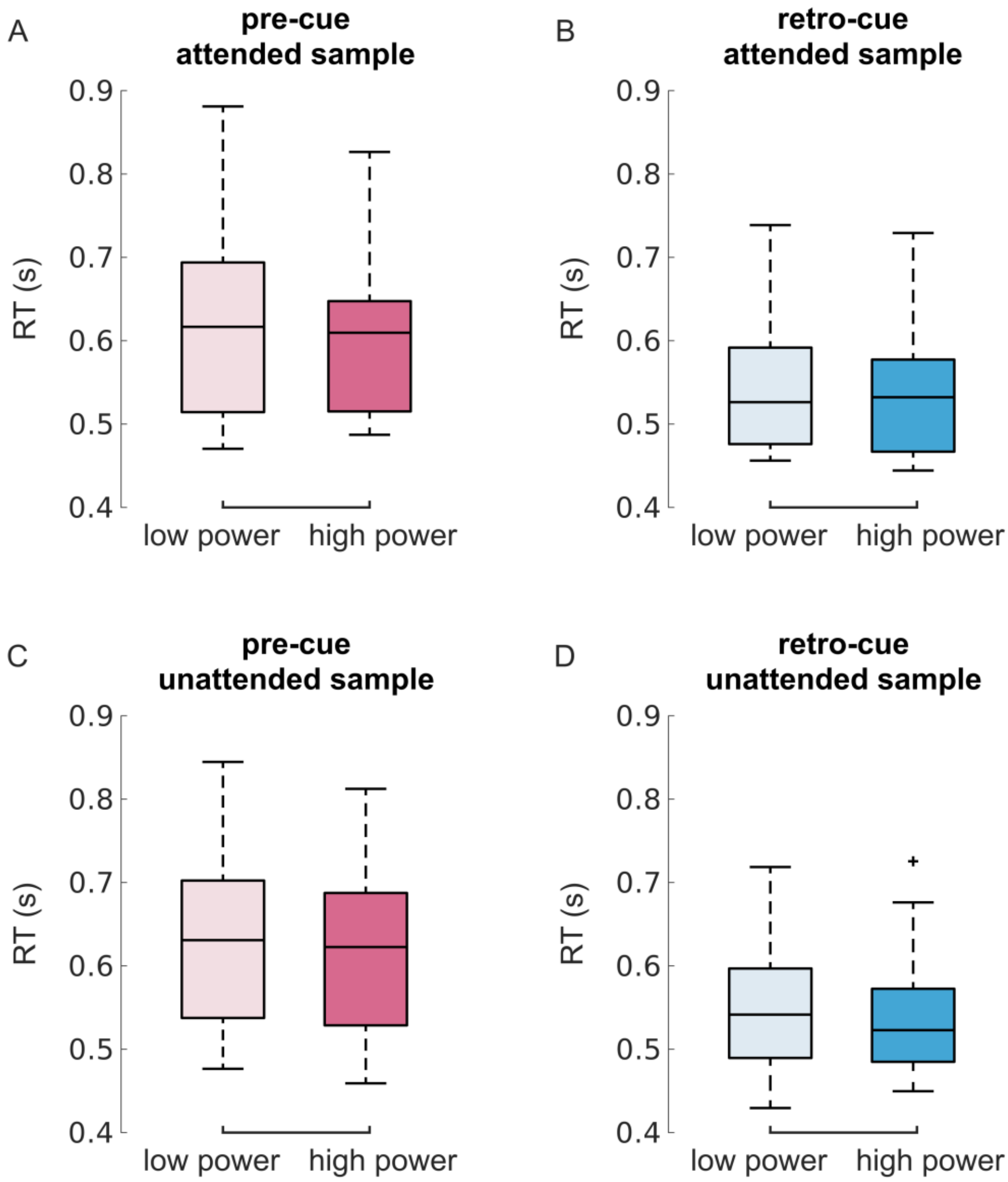

Figure S5. Relationship between pre-stimulus Alpha1 power and RT per task condition.

[A] Effect of pre-stimulus alpha power modulation on reaction times (RT) for the attended sample in the pre-cue condition. RT was compared between low- and high-power trials (median split), showing no significant difference. [B] Same as A for the attended sample in the retro-cue condition. [C] Same as A for the unattended sample in the pre-cue condition. [D] Same as A for the unattended sample in the retro-cue condition. Within each boxplot, the horizontal line represents the median, the box delineates the area between the first and third quartiles (interquartile range).

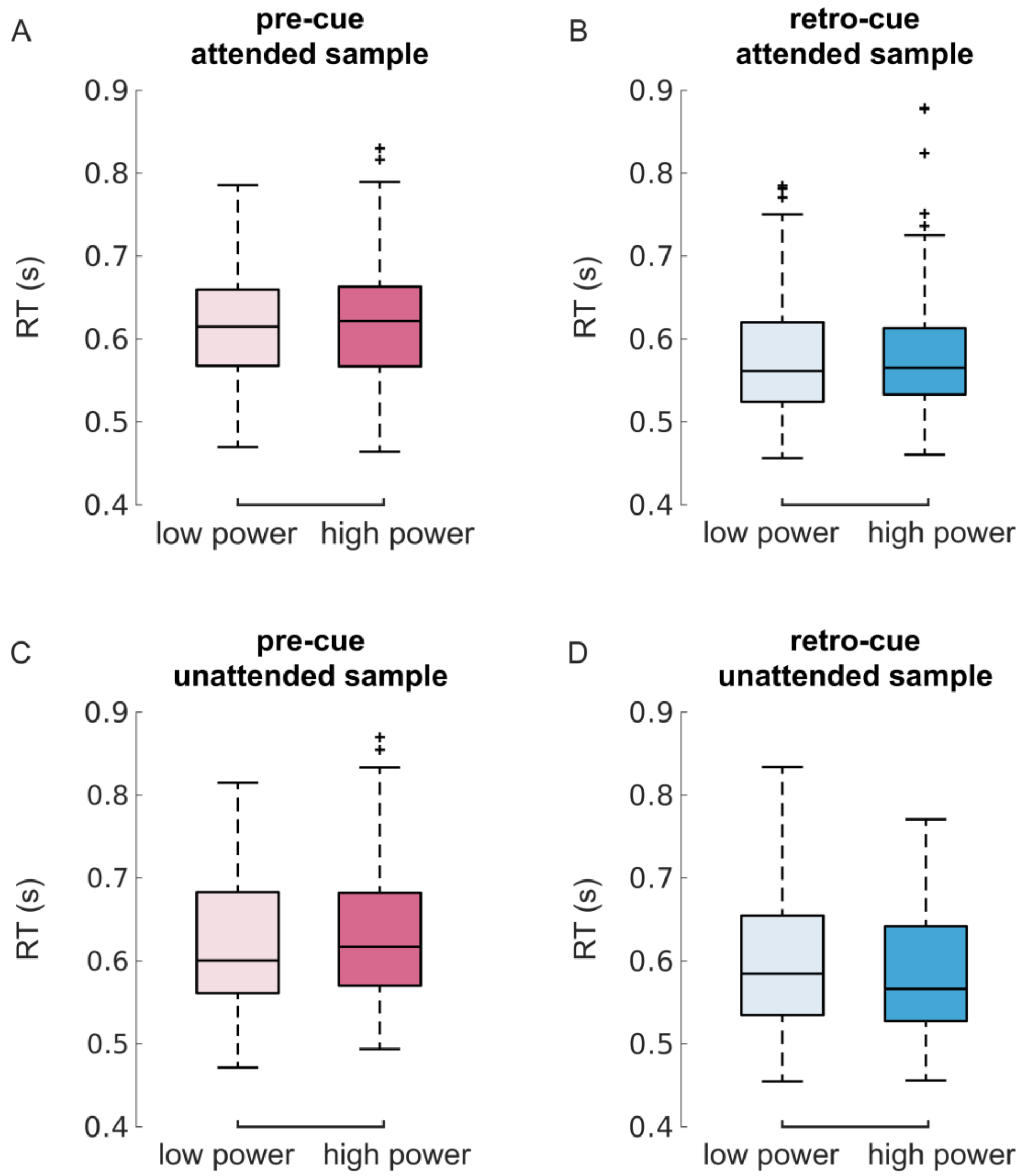

Figure S6. Relationship between pre-stimulus Alpha2 power and RT per task condition.

[A] Effect of pre-stimulus alpha power modulation on reaction times (RT) for the attended sample in the pre-cue condition. RT was compared between low- and high-power trials (median split), showing no significant difference. [B] Same as A for the attended sample in the retro-cue condition. [C] Same as A for the unattended sample in the pre-cue condition. [D] Same as A for the unattended sample in the retro-cue condition. Within each boxplot, the horizontal line represents the median, the box delineates the area between the first and third quartiles (interquartile range).
